# Integrated Multi-Omics Analysis Reveals the Physiological and Metabolic Response Mechanisms of *Luciobarbus capito* Under Cold Stress: Insights from Biochemical Parameters, Gut Microbiota, and Metabolomics

**DOI:** 10.3390/ani16131971

**Published:** 2026-06-26

**Authors:** Kun Guo, Rui Zhang, Haijun Wei, Liang Luo, Shihui Wang, Wei Xu, Nan Sun, Zhigang Zhao

**Affiliations:** 1 Key Open Laboratory of Cold Water Fish Germplasm Resources and Breeding of Heilongjiang Province, Heilongjiang River Fisheries Research Institute, Chinese Academy of Fishery Sciences, Harbin 150070, China; guokun@hrfri.ac.cn (K.G.); zhangrui@hrfri.ac.cn (R.Z.); weihaijun@hrfri.ac.cn (H.W.); luoliang@hrfri.ac.cn (L.L.); wangshihui@hrfri.ac.cn (S.W.); xuwei@hrfri.ac.cn (W.X.); 2 Engineering Technology Research Center of Saline-alkaline Water Fisheries (Harbin), Chinese Academy of Fishery Sciences (CAFS), Harbin 150070, China; 3 College of Hydraulic Science and Engineering, Northeast Agricultural University, Harbin 150030, China

**Keywords:** cold stress, *Luciobarbus capito*, gut microbiota, liver metabolomics, oxidative stress

## Abstract

Cold stress is a key environmental constraint in aquaculture, but integrated host responses linking oxidative status, intestinal microbiota, and hepatic metabolism remain insufficiently characterized in *Luciobarbus capito*. This study aimed to evaluate the biochemical, microbial, and metabolic responses of *L. capito* to acute low-temperature exposure. Fish were exposed to 12 °C for 96 h, with fish maintained at 22 °C as controls; hepatic antioxidant indices, serum biochemical parameters, intestinal microbiota based on 16S rRNA gene sequencing, and liver metabolomic profiles were analyzed. Cold exposure reduced hepatic total superoxide dismutase (T-SOD) and catalase (CAT) activities and increased malondialdehyde (MDA) content, while serum alanine aminotransferase (ALT) and aspartate aminotransferase (AST) activities increased and acid phosphatase (ACP) activity decreased. The intestinal microbiota showed reduced richness and compositional shifts, including decreased Cetobacterium and increased Pseudomonas. Liver metabolomics identified 172 differential metabolites, with enriched pathways related to glycerophospholipid metabolism, α-linolenic acid metabolism, pantothenate and CoA biosynthesis, and ascorbate and aldarate metabolism. Correlation analysis indicated significant associations between altered bacterial genera and hepatic metabolites. These results suggest that acute cold stress disrupts oxidative balance, intestinal microbial composition, and hepatic metabolism in *L. capito*, providing an integrated view of associated physiological and metabolic responses to low-temperature stress.

## Simple Summary

Sudden drops in water temperature are a common problem in fish farming and can harm fish health and production. Aral barbel (*Luciobarbus capito*) is a promising farmed fish, but little is known about how it responds to short periods of cold water. This study examined whether cold exposure affects the liver, blood, gut bacteria, and the small molecules that help the body use energy and protect cells. Fish kept in cold water for four days showed signs of weakened protection against harmful cell damage, increased physiological stress, changes in the balance of gut bacteria, and disturbances in processes related to cell membranes and energy use. These results show that sudden cold exposure affects several connected body systems rather than a single organ. The findings improve our understanding of how Aral barbel copes with low temperature and may help farmers develop better cold-season management strategies, reduce health risks, and support more stable fish production.

## 1. Introduction

Water temperature is a major abiotic factor regulating key physiological processes in aquatic organisms, including growth, immune function, and reproduction [1,2,3]. As ectothermic vertebrates, fish are highly susceptible to thermal fluctuations because each species has a defined thermal tolerance range [4,5]. When water temperature falls below the lower limit of this thermal tolerance range, cold stress can disrupt metabolic homeostasis, impair organ function, and, under severe conditions, cause mortality [6]. Climate variability, particularly the increasing frequency of extreme cold events, poses a growing threat to aquatic ecosystems and aquaculture production, resulting in substantial economic losses [4,7]. Therefore, elucidating the physiological responses of fish to low-temperature stress and their underlying basis is of both ecological and economic importance.

The liver, as a central metabolic organ, is one of the primary targets of cold stress in fish. Previous studies have shown that low-temperature exposure can induce hepatocyte degeneration, metabolic dysfunction, DNA damage, and changes in hepatic gene expression [8,9,10]. Serum biochemical parameters are widely used as sensitive indicators of tissue injury and altered physiological status in fish [11]. The intestine is another key target of environmental stress and plays essential roles in nutrient absorption, immune regulation, and host homeostasis [12,13]. Its resident microbiota contributes substantially to intestinal homeostasis and is highly sensitive to temperature fluctuations [14,15]. Cold exposure may therefore disturb intestinal microbial homeostasis while simultaneously impairing hepatic function. In this context, the gut–liver axis provides a useful framework for understanding inter-organ crosstalk mediated by the bidirectional exchange of metabolites, immune mediators, and microbial products [16,17]. However, most previous studies have examined hepatic and intestinal responses to cold stress separately, and the coordinated changes in gut microbial composition and hepatic metabolism under acute cold exposure remain poorly characterized.

Aral barbel (*Luciobarbus capito*) is a euryhaline cyprinid species introduced into China from Uzbekistan in 2003 and has since become an important aquaculture fish because of its rapid growth, omnivorous feeding habit, and broad environmental adaptability [18]. The optimum growth temperature for *L. capito* ranges from 24 °C to 27 °C [19], suggesting that exposure to lower temperatures may impose considerable physiological stress. Previous studies on *L. capito* have mainly focused on aquaculture practices, saline–alkali tolerance, and general biological characteristics [20,21]. However, its physiological and metabolic responses to acute cold stress remain largely unexplored. In the present study, we combined serum biochemical analysis, 16S rRNA gene sequencing of the gut microbiota, and liver metabolomics to investigate cold stress-induced physiological and metabolic disturbances in *L. capito* and to examine the potential associations between intestinal microbial dysbiosis and hepatic metabolic alteration. This integrative approach was intended to provide a broader understanding of the biochemical and physiological responses of fish to acute low-temperature stress and to explore potential associations between intestinal microbial changes and hepatic metabolic alterations within a gut–liver interaction framework.

## 2. Materials and Methods

### 2.1. Experimental Fish

Healthy one-year-old *L. capito* with a mean body weight of 30.85 ± 2.62 g were obtained from the Hulan Experimental Station of the Heilongjiang River Fisheries Research Institute, Chinese Academy of Fishery Sciences. Before the experiment, the fish were acclimated for 7 days under the following conditions: water temperature, 22 ± 1 °C; pH, 7.8 ± 0.2; dissolved oxygen (DO), >6.5 mg/L; and a 12 h light/12 h dark photoperiod. During acclimation, the fish were fed a commercial diet twice daily at 08:00 and 17:00. Fish were fasted for 24 h before the cold stress trial to minimize the confounding effects of digestive activity on physiological measurements.

### 2.2. Experimental Design, Low-Temperature Challenge, and Sampling

After acclimation, the fish were randomly assigned to two groups: a control group (CG, 22 °C) and a low-temperature stress group (LT, 12 °C), with six replicate tanks per group (*n* = 15 fish per tank). In the LT group, the water temperature was gradually reduced from the acclimation temperature (22 °C) to the target temperature (12 °C) at a rate of 1 °C/h and then maintained at 12 °C for 96 h. The CG group was maintained at 22 °C throughout the experimental period. Replicate tanks were considered the experimental units for treatment allocation and environmental control.

Sampling began when the water temperature in the LT group reached 12 °C, which was defined as 0 h. At 0, 6, 12, 24, 48, 72, and 96 h, three fish per treatment group were randomly selected for biochemical analysis, with each fish collected from a different replicate tank. At each sampling time point, the fish were anesthetized with MS-222, and blood was collected from the caudal vein. For serum preparation, blood samples were kept at 4 °C for 3 h, centrifuged at 3000 rpm for 10 min at 4 °C, and the resulting serum was stored at −80 °C. Liver tissue samples were excised, immediately frozen in liquid nitrogen, and stored at −80 °C for antioxidant enzyme assays. At the end of the 96 h exposure period, liver samples were collected from six fish per group for metabolomics analysis, and intestinal tissues were collected from five fish per group for gut microbiota analysis.

### 2.3. Serum Biochemistry and Hepatic Antioxidant Assays

Approximately 0.1 g of liver tissue was placed in a 2 mL tube containing nine volumes of 0.86% saline solution and homogenized under low-temperature conditions using a Scienta-48L cryogenic high-throughput tissue homogenizer. The homogenate was centrifuged at 2500 rpm for 10 min at 4 °C, and the resulting supernatant was collected for antioxidant enzyme assays.

Serum activities of metabolic enzymes, including alanine aminotransferase (ALT), aspartate aminotransferase (AST) and acid phosphatase (ACP), were measured using a fully automated biochemical analyzer (BS200, Mindray, Shenzhen, China) with the corresponding reagent kits. Hepatic antioxidant and oxidative stress parameters, including catalase (CAT) activity, total superoxide dismutase (T-SOD) activity, and malondialdehyde (MDA) content, were determined using commercial assay kits (Nanjing Jiancheng Bioengineering Institute, Nanjing, China) according to the manufacturer’s instructions.

Biochemical data are presented as the mean ± standard deviation (SD). Before statistical comparisons, data normality was assessed using the Shapiro–Wilk test, and homogeneity of variance was evaluated using Levene’s test. Differences among sampling time points were analyzed by one-way analysis of variance (ANOVA) using SPSS version 19.0, followed by Duncan’s multiple range test where applicable, and *p* < 0.05 was considered statistically significant.

### 2.4. Intestinal Microbiota Analysis

Microbial genomic DNA was extracted from intestinal samples using the DNeasy^®^ PowerSoil^®^ Pro Kit (QIAGEN, Hilden, Germany) according to the manufacturer’s instructions. DNA quality was assessed by 1% agarose gel electrophoresis, and DNA concentration and purity were determined using a NanoDrop 2000 spectrophotometer (Thermo Scientific, Waltham, MA, USA). For each sample, 10 ng of qualified genomic DNA was used for library preparation and subsequent sequencing. The V3–V4 hypervariable region of the 16S rRNA gene was amplified using primers 338F (5′-ACTCCTACGGGAGGCAGCAG-3′) and 806R (5′-GGACTACHVGGGTWTCTAAT-3′). After purification, sequencing libraries were prepared using the NEXTFLEX Rapid DNA-Seq Kit and subjected to paired-end sequencing on an Illumina MiSeq PE300 platform (Majorbio Bio-Pharm Technology Co., Ltd., Shanghai, China). Raw sequencing reads were quality-filtered using fastp (version 0.19.6) and paired-end reads were merged using FLASH (version 1.2.7). High-quality sequences were clustered into operational taxonomic units (OTUs) at 97% sequence similarity using UPARSE (version 7.1), with chimeric sequences identified and removed during the clustering process. Alpha-diversity indices, including Chao1, Shannon, Simpson, and ACE, were calculated using Mothur software (v1.48.3) package and compared between the CG and LT groups using the Wilcoxon rank-sum test. A Venn diagram was generated to visualize shared and unique OTUs. Beta-diversity was assessed via principal coordinate analysis (PCoA) based on Bray–Curtis distances, and group differences were tested using analysis of similarities (ANOSIM). Microbial community composition was analyzed at the phylum and genus levels.

### 2.5. Liver Metabolomics

Liver tissue samples (50 mg) were weighed and homogenized in 400 µL of methanol/water (4:1, *v*/*v*) using a high-throughput tissue homogenizer (Wonbio-96c, Wonbio, Shanghai, China) at 50 Hz for 6 min. The homogenate was vortexed for 30 s and sonicated at 40 kHz for 30 min at 5 °C. To precipitate proteins, the mixture was incubated at −20 °C for 30 min and then centrifuged at 13,000× *g* for 15 min at 4 °C. The supernatant was transferred to injection vials for LC-MS/MS analysis. A pooled quality control (QC) sample was prepared by combining equal aliquots of all individual samples.

Untargeted metabolomic profiling was performed by Shanghai Majorbio Bio-pharm Technology Co., Ltd. (Shanghai, China) using an ultra-high-performance liquid chromatography system coupled to a Q Exactive mass spectrometer (UHPLC-Q Exactive, Thermo Fisher Scientific, Waltham, MA, USA). Chromatographic separation was achieved on a Waters HSS T3 column (Waters, Milford, MA, USA, 100 mm × 2.1 mm i.d., 1.8 μm). The injection volume was 3 μL. Mobile phase A consisted of water/acetonitrile (95:5, *v*/*v*) containing 0.1% formic acid, and mobile phase B consisted of acetonitrile/isopropanol/water (47.5:47.5:5, *v*/*v*/*v*) containing 0.1% formic acid. The flow rate was 0.40 mL/min, and the column temperature was maintained at 40 °C. Mass spectrometric data were acquired in both positive and negative ion modes over a scan range of *m*/*z* 70–1050. The electrospray ionization parameters were as follows: spray voltage, 3500 V in positive mode and −3000 V in negative mode; sheath gas, 50 arb; auxiliary gas, 13 arb; ion source temperature, 450 °C; and stepped collision energy, 20, 40, and 60 V.

The raw LC-MS data were imported into Progenesis QI software (v3.0, Waters Corporation, Milford, MA, USA) for baseline filtering, peak detection, integration, retention time correction, and peak alignment. A data matrix containing retention time, mass-to-charge ratio, and peak intensity was generated. Metabolites were annotated by matching MS and MS/MS information against public databases, including the Human Metabolome Database (HMDB) and METLIN, as well as the Majorbio in-house database. The data matrix was then uploaded to the Majorbio Cloud Platform for further analysis. Variables with non-zero values in less than 80% of samples in at least one group were removed according to the 80% rule. Missing values were replaced with the minimum value in the original matrix. Peak intensities were normalized using the total ion current normalization method to reduce variation caused by sample preparation and instrumental instability. Variables with a relative standard deviation (RSD) > 30% in QC samples were removed, and the resulting data were log10-transformed before multivariate statistical analysis.

Multivariate statistical analyses, including principal component analysis (PCA) and orthogonal partial least squares-discriminant analysis (OPLS-DA), were performed using the ropls package (v1.6.2) in R (v1.0.0) The OPLS-DA model was validated using a 200-permutation test. Differential metabolites (DMs) were identified using a combined criterion of variable importance in projection (VIP) > 1.0 from the OPLS-DA model and FDR-adjusted *p* < 0.05 based on the Benjamini–Hochberg correction. Differential metabolites were subjected to metabolic pathway enrichment analysis using the Kyoto Encyclopedia of Genes and Genomes (KEGG) database.

### 2.6. Correlation Analysis

Pearson correlation analysis was performed to assess correlations between intestinal bacterial genera and hepatic differential metabolites. Significance levels are defined as * *p* < 0.05, ** *p* < 0.01 and *** *p* < 0.001. A heatmap was generated to visualize the correlation matrix between significantly altered intestinal bacterial genera and hepatic DMs.

## 3. Results

### 3.1. Changes in Biochemical Indices

**Figure 1:**
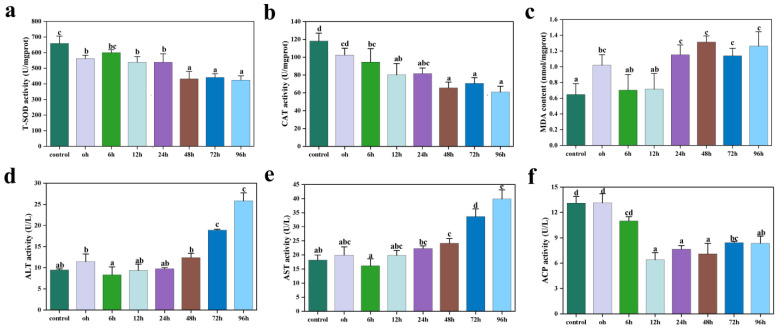
Effects of acute cold stress on biochemical indices in *L. capito*. (**a**–**c**) Hepatic indices: total superoxide dismutase (T-SOD) activity, catalase (CAT) activities, and malondialdehyde (MDA) content. (**d**–**f**) Serum indices: alanine aminotransferase (ALT), aspartate aminotransferase (AST), and acid phosphatase (ACP) activities. Data are presented as mean ± SD (*n* = 3 fish per group). Different lowercase letters indicate statistically significant differences among groups (*p* < 0.05; one-way analysis of variance (ANOVA) followed by Duncan’s multiple range test).

Acute cold stress significantly altered multiple biochemical indices in *L. capito*, including hepatic T-SOD and CAT activities and MDA content, as well as serum ALT, AST, and ACP activities (Figure 1). T-SOD activity was significantly suppressed throughout the exposure period, reached its lowest level during 48–96 h, and remained significantly lower than that in the control group at all sampling time points (*p* < 0.05; Figure 1a). CAT activity decreased after cold exposure and remained relatively low from 12 h onward, with values significantly lower than those in the control group until the end of the experiment (*p* < 0.05; Figure 1b). MDA content increased significantly by 24 h and remained elevated thereafter (24–96 h; *p* < 0.05; Figure 1c). ALT and AST activities remained relatively stable during the early stage of exposure and increased significantly at later time points (*p* < 0.05; Figure 1d,e). In contrast, ACP activity decreased sharply at 12 h and remained low throughout the rest of the exposure period (*p* < 0.05; Figure 1f).

### 3.2. Intestinal Microbiota Changes

#### 3.2.1. Richness and Diversity

**Figure 2:**
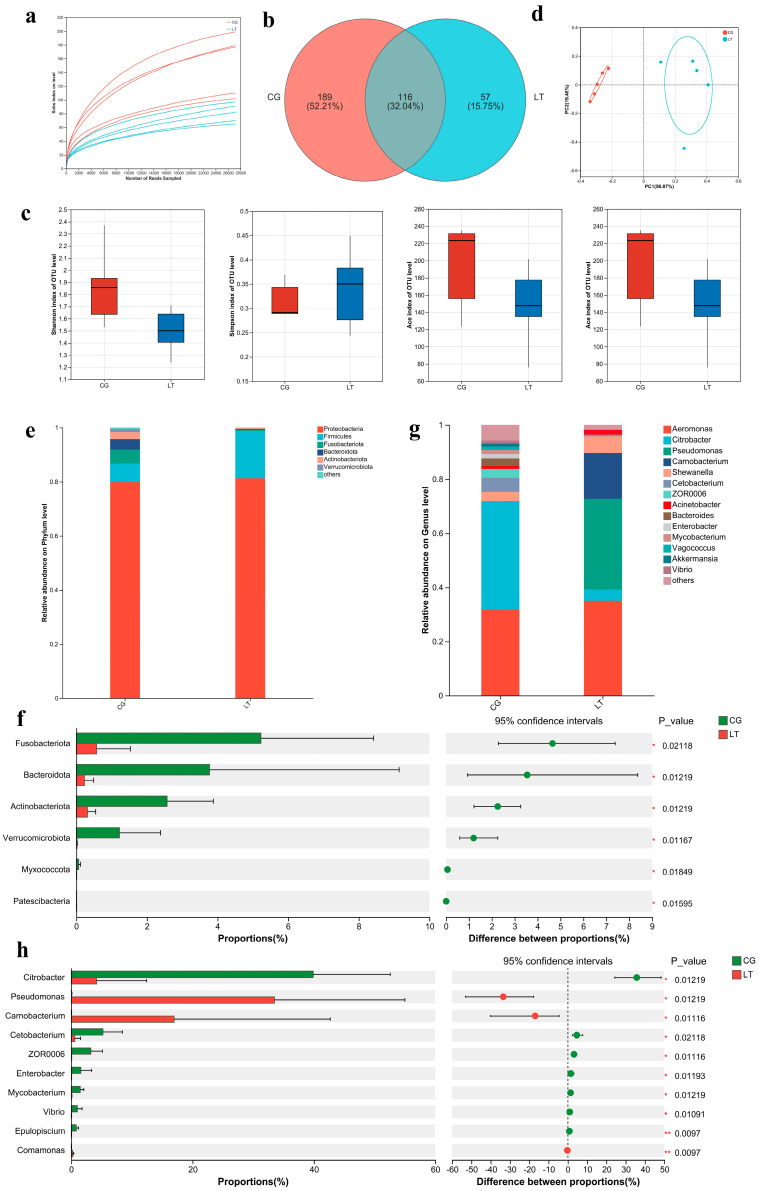
Effects of cold stress on the gut microbiota of *L. capito*. (**a**) Rarefaction curves. (**b**) Venn diagram showing shared and unique operational taxonomic units (OTUs). (**c**) Alpha-diversity indices (Shannon, Simpson, ACE, and Chao1). (**d**) Principal coordinate analysis (PCoA) based on Bray–Curtis distances. (**e**) Community composition at the phylum level. (**f**) Differential abundance analysis at the phylum level. (**g**) Community composition at the genus level. (**h**) Differential abundance analysis at the genus level. Intestinal microbiota analysis was performed using five biological replicates per group (*n* = 5). *, and ** indicate significant correlations at *p* < 0.05, and *p* < 0.01, respectively.

The rarefaction curves for all samples approached a plateau, indicating that the sequencing depth was sufficient to capture the most intestinal microbial diversity (Figure 2a). Venn diagram analysis showed that the two groups shared 116 OTUs, whereas 57 and 189 OTUs were unique to the LT and control groups, respectively (Figure 2b). Alpha-diversity analysis was performed to compare the richness and diversity of the intestinal microbiota between the CG and LT groups (Figure 2c). Compared with the control group, the LT group showed a significantly lower Chao1 index (*p* < 0.05), whereas no significant differences were observed in the Shannon, Simpson, and ACE indices. Principal coordinate analysis (PCoA) based on Bray–Curtis distances showed separation between the microbial communities of the two groups (Figure 2d). This difference in community structure was further supported by analysis of similarities (ANOSIM) (*p* = 0.004).

#### 3.2.2. Relative Abundance of the Intestinal Microbiota

At the phylum level, the intestinal microbiota of both groups was dominated by Proteobacteria and Firmicutes (Figure 2e). In the control group, Proteobacteria and Firmicutes accounted for 79.88% and 6.84% of the total community, respectively, whereas in the LT group, they accounted for 81.29% and 17.42%, respectively. Several subdominant phyla showed significantly lower relative abundances in the LT group than in the control group, including Fusobacteriota (5.23% vs. 0.57%), Bacteroidota (3.77% vs. 0.23%), Actinobacteriota (2.57% vs. 0.31%), and Verrucomicrobiota (1.22% vs. 0.02%) (*p* < 0.05; Figure 2f). At the genus level, the control group was dominated by Citrobacter (39.89%), Aeromonas (31.93%), and Cetobacterium (5.23%) (Figure 2g). In the LT group, the intestinal microbiota was dominated by Aeromonas (35.09%), Pseudomonas (33.51%), and Carnobacterium (16.95%). Compared with the control group, the LT group showed a significant decrease in the relative abundance of genera such as Citrobacter and Cetobacterium, together with a significant increase in Pseudomonas and Carnobacterium (*p* < 0.05; Figure 2h).

### 3.3. Metabolomic Analysis

**Figure 3:**
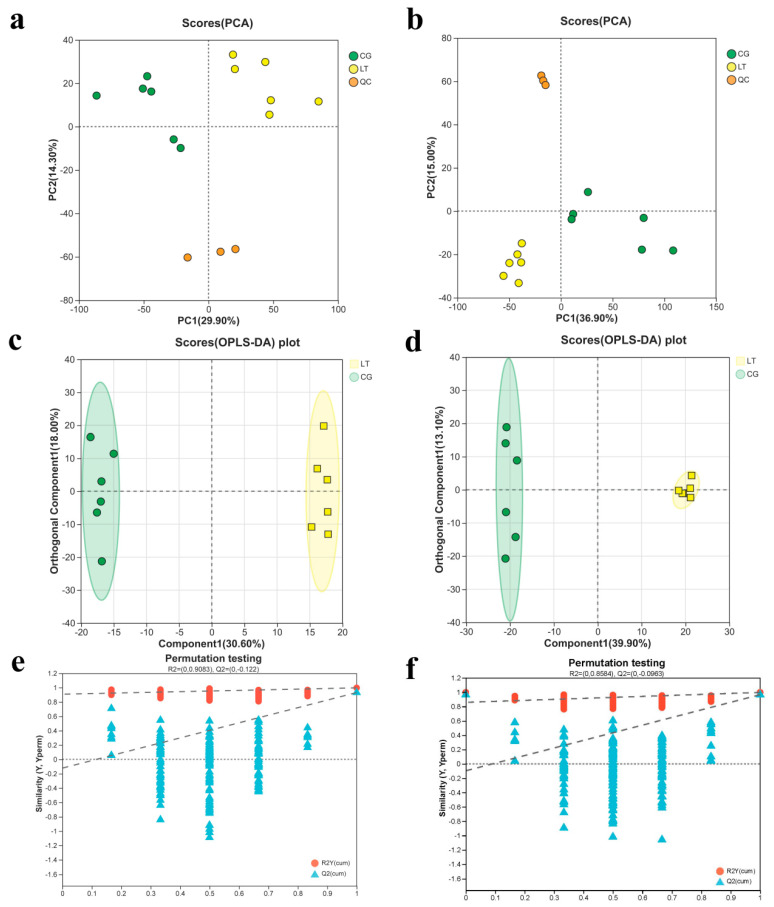
Multivariate analysis of the liver metabolome of *L. capito* under cold stress. (**a**,**b**) Principal component analysis (PCA) score plots in positive and negative ion modes, respectively. (**c**,**d**) Orthogonal partial least squares-discriminant analysis (OPLS-DA) score plots in positive and negative ion modes, respectively. (**e**,**f**) Validation of the OPLS-DA models by 200-permutation tests in positive and negative ion modes, respectively. Liver metabolomic analysis was performed using six biological replicates per group (*n* = 6).

Multivariate statistical analysis revealed distinct liver metabolite profiles between *L. capito* exposed to cold stress and control group. In both positive and negative ion modes, PCA score plots showed tight clustering of the quality control (QC) samples, indicating good analytical stability and reproducibility (Figure 3a,b). OPLS-DA score plots further showed a clear separation between the CG and LT groups in both ionization modes (Figure 3c,d). A 200-permutation test was performed to validate the OPLS-DA models. The permutation results showed that all permuted R^2^ and Q^2^ values were lower than the corresponding original values, and the Q^2^ regression intercepts were below zero, indicating that the models were not overfitted (Figure 3e,f).

**Figure 4:**
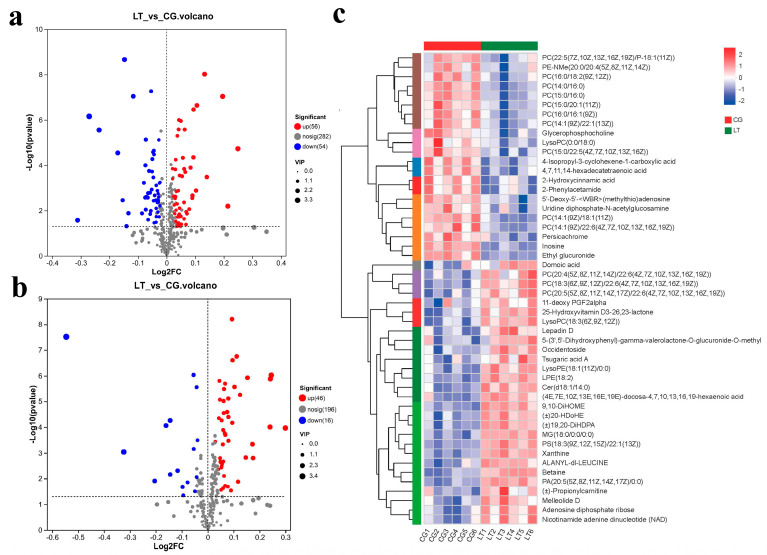
Analysis of differential metabolites in *L. capito* liver under cold stress. (**a**,**b**) Volcano plots showing significantly altered metabolites in positive and negative ion modes, respectively. Points are colored by regulation status: red, upregulated; blue, downregulated; gray, non-significant. (**c**) Heatmap of significantly altered metabolites. The color gradient from red to blue indicates relative abundance from high to low.

Liver metabolic profiles of the CG and LT groups were further analyzed by LC–MS. A total of 172 significantly altered metabolites were identified including 110 in the positive ion mode and 62 in the negative ion mode. In the positive ion mode, 54 metabolites were upregulated and 56 were downregulated, whereas in the negative ion mode, 46 were upregulated and 16 were downregulated. The distribution of significantly altered metabolites is shown in the volcano plots, and their relative abundance patterns are presented in the heatmap (Figure 4). Table 1 lists representative differential metabolites assigned to five KEGG compound classes, including lipids, nucleic acids, peptides, steroids, and vitamins and cofactors.

**Figure 5:**
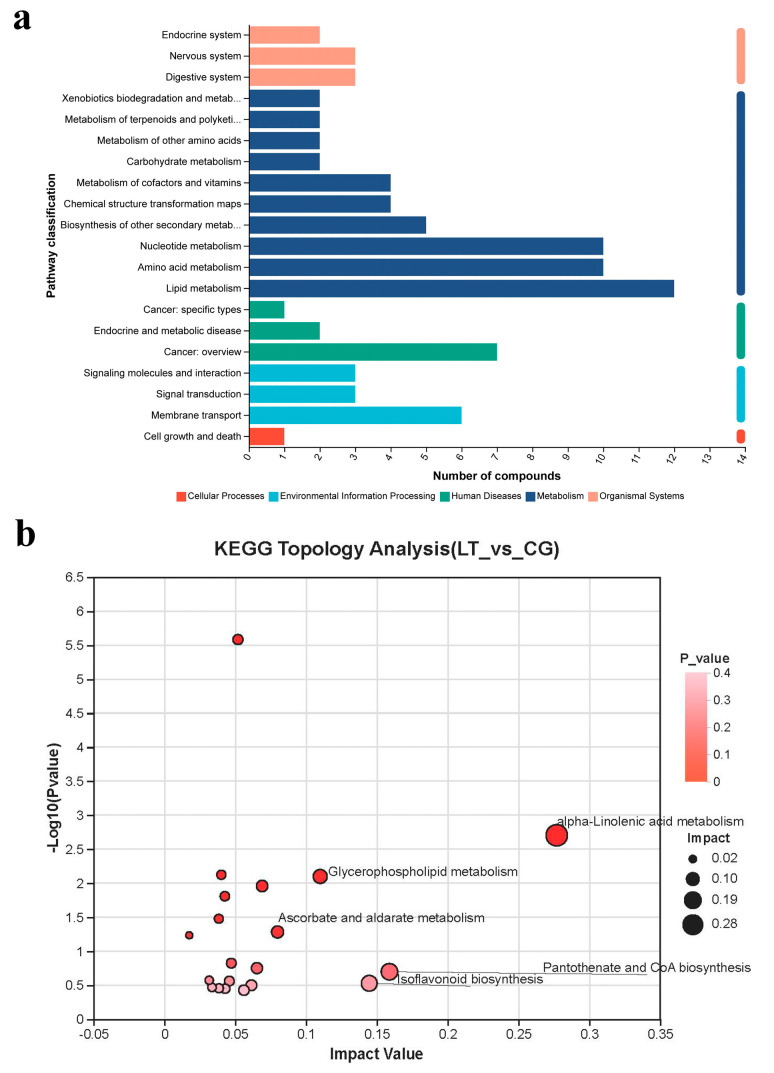
Metabolic pathways analysis of liver metabolism in *L. capito* under cold stress. (**a**) Kyoto Encyclopedia of Genes and Genomes (KEGG) pathway enrichment analysis. (**b**) Pathway topology analysis showing the impact of enriched pathways.

KEGG enrichment and pathway analysis were performed on the identified differential metabolites. These differential metabolites were mainly annotated into five major KEGG level-1 categories: Cellular Processes, Environmental Information Processing, Human Diseases, Metabolism, and Organismal Systems (Figure 5a). Based on the −log10(P) values and pathway impact scores, the major enriched pathways included α-Linolenic acid metabolism, pantothenate and CoA biosynthesis, isoflavonoid biosynthesis, glycerophospholipid metabolism, and ascorbate and aldarate metabolism (Figure 5b).

### 3.4. Association Between the Metabolome and Intestinal Microbiota

**Figure 6:**
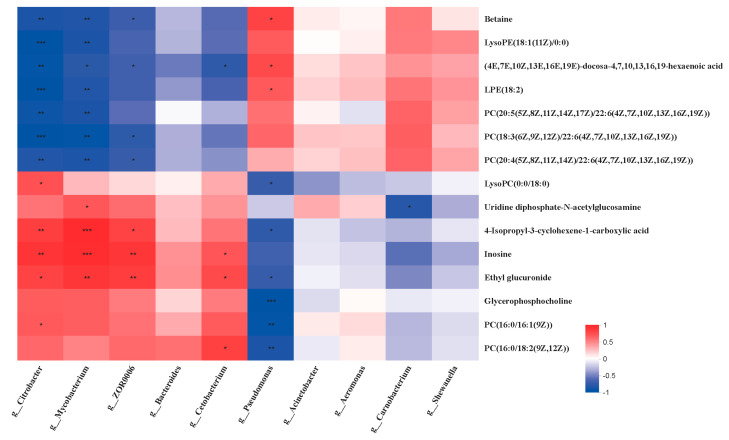
Correlation analysis between gut microbiota and hepatic metabolism in *L. capito* under cold stress. *, **, and *** indicate significant correlations at *p* < 0.05, *p* < 0.01, and *p* < 0.001, respectively.

Pearson correlation analysis was performed to assess the associations between intestinal bacterial genera and liver differential metabolites, and the results are shown in the heatmap (Figure 6). Several altered bacterial genera showed significant correlations with specific liver metabolites. For example, Citrobacter abundance was positively correlated with LysoPC(0:0/18:0), 4-Isopropyl-3-cyclohexene-1-carboxylic acid, inosine, ethyl glucuronide and PC(16:0/16:1(9Z)). Conversely, Citrobacter abundance was negatively correlated with PC(20:5(5Z,8Z,11Z,14Z,17Z)/22:6(4Z,7Z,10Z,13Z,16Z,19Z)), Betaine, LysoPE(18:1(11Z)/0:0) and PC(18:3(6Z,9Z,12Z)/22:6(4Z,7Z,10Z,13Z,16Z,19Z)). Similarly, Pseudomonas abundance was positively correlated with betaine but negatively with LysoPC(0:0/18:0), ethyl glucuronide, and PC(16:0/18:2(9Z,12Z)).

## 4. Discussion

Climate variability is increasing the frequency and intensity of extreme cold events, posing an important challenge to aquaculture production. Although the adverse effects of cold stress on fish physiology have been widely recognized, the integrated responses linking oxidative status, intestinal microbiota, and hepatic metabolism remain insufficiently understood. In the present study, we combined biochemical assays, gut microbiota profiling, and liver metabolomics to investigate the response of *L. capito* to acute cold exposure. The results indicate that cold stress induced coordinated physiological and metabolic disturbances, including hepatic oxidative imbalance, serum enzyme perturbation, intestinal microbial restructuring, and liver metabolic alteration. Collectively, these findings suggest a potential association between host metabolic responses and gut microbial changes under cold stress, consistent with the involvement of gut–liver interactions.

### 4.1. Effect of Cold Stress on Hepatic Biochemical Responses in L. capito

Oxidative stress is a major mechanism of physiological injury in fish exposed to environmental stressors and arises from an imbalance between oxidant generation and antioxidant defenses [22]. Under normal conditions, reactive oxygen species (ROS) production and clearance remain in dynamic equilibrium, whereas environmental stress can disrupt this balance and promote oxidative damage to lipids, proteins, and nucleic acids [23]. In the present study, acute cold stress markedly reduced hepatic T-SOD and CAT activity while increasing MDA content, indicating that the antioxidant defense system of *L. capito* was impaired under low-temperature exposure.

SOD and CAT are key enzymatic components of the antioxidant system and play central roles in limiting ROS accumulation [24]. Their sustained suppression under cold stress suggests that hepatic antioxidant capacity was insufficient to maintain redox homeostasis. The subsequent increase in MDA, a terminal product of lipid peroxidation, further indicates enhanced oxidative damage to cellular membranes [25]. Notably, the decline in antioxidant enzyme activities preceded or accompanied the elevation of MDA, a pattern consistent with progressive oxidative injury reported in other cold-stressed aquatic organisms [26,27]. Together, these findings support the conclusion that acute cold exposure induced oxidative stress in the liver of *L. capito*.

Serum biochemical parameters also provided evidence of physiological disturbance under cold stress. ALT and AST are commonly used indicators of tissue injury and altered membrane integrity in fish [28,29]. Their elevation in the LT group suggests that acute cold exposure compromised tissue function and metabolic stability. In contrast, ACP activity in the LT group decreased significantly from 6 h onward. Because ACP is associated with lysosomal function and non-specific immune responses in aquatic animals [30], this decline may reflect impaired lysosomal enzyme activity and weakened physiological defense under low-temperature conditions. Similar decreases in ACP activity have also been reported in cold-stressed *Thunnus albacares* [27]. Overall, the combined biochemical responses indicate that acute cold stress may have affected tissue function and metabolic stability. The biochemical assays in the present study were performed with three biological replicates at each sampling time point, a replication level commonly used in controlled aquatic stress experiments. This design allowed the detection of major physiological responses to acute cold stress; nevertheless, future studies with larger sample sizes would be useful to further validate these findings and better characterize inter-individual variation. As the present study focused primarily on biochemical and omics-based responses, histological evaluation of hepatic structural changes would provide additional evidence for further understanding the hepatic effects of acute cold stress.

### 4.2. Effect of Cold Stress on the Gut Microbiota of L. capito

The intestine is an important immune organ, and its resident microbiota contributes substantially to host homeostasis and health [31,32]. Because intestinal microbial communities are sensitive to environmental change, temperature shifts can substantially alter microbial richness and community structure [33,34]. In the present study, acute cold stress significantly reduced the Chao1 index and decreased the number of unique OTUs in the intestinal microbiota of *L. capito*, indicating a contraction in microbial richness. Because microbial richness is generally associated with community stability and ecological resilience, this reduction may reflect a less stable intestinal microbial community under cold stress [35]. Notably, the Shannon and Simpson indices did not differ significantly between groups, suggesting that cold exposure affected richness more strongly than overall evenness or dominance structure. Together, these findings indicate that acute cold stress simplified the intestinal microbial community of *L. capito*, particularly by reducing rare or low-abundance taxa, while leaving the dominant community framework relatively preserved.

At the phylum level, Proteobacteria and Firmicutes remained dominant in both groups, which is consistent with reports for many fish species [36,37]. Firmicutes have been linked to microbial processes involved in host energy metabolism, including short-chain fatty acid production and lipid-related metabolic functions [38]. Accordingly, the increase in Firmicutes observed here may reflect cold stress-induced restructuring of the intestinal microbiota, with potential implications for host energy homeostasis. However, because the gut microbiota analysis in the present study was based on 16S rRNA gene sequencing, this result should be interpreted as a taxonomic shift with potential functional implications rather than direct evidence of altered microbial energy metabolism.

At the genus level, the marked reduction in Cetobacterium is noteworthy because this taxon is widely regarded as a beneficial member of the fish intestinal microbiota and has been associated with vitamin B_12_ production and metabolic support [39]. The decrease in Cetobacterium observed here may therefore suggest a potential reduction in beneficial microbial taxa under cold stress. By contrast, the increased abundance of Pseudomonas suggests an enrichment of opportunistic taxa and a less stable intestinal microbial state. Similar increases in Pseudomonas have been reported in stressed aquatic animals [40,41]. Nevertheless, in the absence of direct pathogen or inflammation data, this shift should be interpreted as evidence of dysbiosis rather than proof of infection. Taken together, these results indicate that acute cold stress not only reduced microbial richness but also promoted compositional restructuring of the intestinal microbiota in *L. capito*.

### 4.3. Effect of Cold Stress on Liver Metabolism in L. capito

Metabolomic analysis revealed pronounced hepatic metabolic alteration in *L. capito* under acute cold stress, with 110 and 62 differential metabolites identified in the positive and negative ion modes, respectively. This result indicates that cold exposure triggered broad changes in liver metabolism rather than a uniform suppression of metabolic activity. Similar metabolome-wide responses have been reported in fish exposed to environmental stressors such as hypoxia and temperature fluctuation, suggesting that metabolic plasticity is a central component of stress adaptation in ectothermic vertebrates [42,43,44,45].

Among the altered pathways, lipid-related metabolism was particularly prominent, especially glycerophospholipid metabolism and alpha-linolenic acid metabolism. Membrane phospholipids are essential for maintaining membrane integrity, fluidity, and signaling capacity, and are therefore highly sensitive to temperature change [46,47]. In the present study, several PCs were decreased whereas multiple LysoPCs were increased in the LT group, suggesting enhanced phospholipid turnover and changes in membrane lipid composition under cold stress. LysoPCs are generated through phospholipid hydrolysis and are often regarded as indicators of membrane remodeling and stress-related lipid turnover [48]. Therefore, the observed shift from PCs to LysoPCs may reflect a compensatory response aimed at preserving membrane-associated functions under low-temperature conditions. Such a pattern is consistent with the concept of homeoviscous adaptation, whereby ectothermic animals modify membrane lipid composition to maintain cellular stability during thermal stress [42,49]. Furthermore, the enrichment of glycerophospholipid metabolism was primarily associated with the differential accumulation of several VIP-selected phospholipid metabolites, including PC(15:0/16:0), PC(16:0/16:0), and multiple LysoPC species. These metabolites likely contributed substantially to the pathway impact observed in Figure 5 and support the occurrence of membrane lipid remodeling under cold stress.

Cold stress also altered metabolites associated with energy demand and cofactor metabolism. The enrichment of pantothenate and CoA biosynthesis suggests that cold exposure-affected pathways related to cofactor supply and intermediary metabolism, as CoA is essential for fatty acid oxidation, the tricarboxylic acid cycle, and multiple biosynthetic processes [50]. Consistent with this pathway enrichment, D-pantothenic acid was identified as a significant differential metabolite with a VIP value greater than 1.0, further supporting the involvement of cofactor metabolism in the hepatic response to cold stress. In parallel, changes in nucleotide-related metabolites indicate a disturbance in hepatic metabolic homeostasis and may reflect altered energetic demand and metabolic turnover under cold stress [51,52]. The elevated cortisol level observed in the LT group is also consistent with the occurrence of systemic stress and enhanced metabolic mobilization, as cortisol is a major endocrine mediator of energy redistribution in stressed fish [53,54]. Together, these results suggest that acute cold stress was associated with marked metabolic disturbance in the liver and with changes in pathways involved in energy utilization and biochemical support. These changes further suggest a possible shift in hepatic energy strategy under cold stress. Under low-temperature exposure, energy metabolism may be redirected from routine growth- and maintenance-related processes toward stress adaptation, including membrane remodeling, antioxidant defense, and cellular repair. The involvement of pantothenate and CoA biosynthesis indicates that fatty acid oxidation and TCA cycle-related metabolism may be affected, because CoA serves as a key carrier for acyl groups and acetyl-CoA generation. Meanwhile, the altered phospholipid and α-linolenic acid-related metabolites suggest that lipid-derived substrates may contribute not only to membrane adaptation but also to energy supply under cold stress.

In addition to lipid and energy metabolism, antioxidant-related metabolic responses were also evident. Ascorbate and aldarate metabolism was significantly enriched, and ascorbic acid showed a decreasing trend in the LT group. Ascorbic acid was also identified as a VIP-selected metabolite, indicating that the enrichment of this pathway was closely linked to alterations in antioxidant-related metabolites under cold stress. Ascorbic acid is an important non-enzymatic antioxidant involved in ROS scavenging and maintenance of redox balance, and its depletion is often associated with increased oxidative challenge [55,56]. This metabolomic signature is consistent with the biochemical results, which showed reduced hepatic T-SOD and CAT activity together with elevated MDA content. Therefore, the metabolomic and biochemical data collectively support the view that oxidative imbalance was an important component of the hepatic response of *L. capito* to acute cold stress. Overall, these findings suggest that the liver of *L. capito* responds to low-temperature exposure through coordinated metabolic adjustment involving membrane lipid alteration, altered energy-related metabolism, and increased antioxidant demand.

From a physiological and aquaculture perspective, these pathway-level changes provide further insight into the adaptive and potentially detrimental responses of *L. capito* to acute cold exposure. The enrichment of glycerophospholipid metabolism and α-linolenic acid metabolism suggests that membrane lipid remodeling is a key physiological response to low temperature, which may help maintain membrane fluidity and cellular stability in ectothermic fish. The involvement of pantothenate and CoA biosynthesis indicates that hepatic energy metabolism and cofactor supply may be altered to meet the increased energetic demand for stress adaptation. Meanwhile, the enrichment of ascorbate and aldarate metabolism is consistent with the observed oxidative imbalance, suggesting increased utilization of antioxidant-related metabolites under cold stress. These pathway-level responses may help explain the biochemical changes observed in this study, including reduced antioxidant enzyme activities, increased lipid peroxidation, and altered serum enzyme activities. From an aquaculture viewpoint, these findings suggest that sudden low-temperature exposure may compromise liver function and metabolic stability in *L. capito*. Therefore, reducing abrupt temperature fluctuations and optimizing nutritional strategies related to lipid quality, energy supply, and antioxidant support may be beneficial for improving cold-stress tolerance during cold-season culture.

### 4.4. Correlation Between Liver Metabolome and Intestinal Microbiota

Correlation analysis revealed significant associations between altered intestinal bacterial genera and hepatic differential metabolites in *L. capito*, suggesting that intestinal microbial restructuring under cold stress was associated with hepatic metabolic alteration. In particular, Citrobacter and Pseudomonas showed contrasting correlations with betaine and several metabolites related to phospholipid metabolism. Given that betaine is an important osmolyte [57] and phospholipid metabolism is closely related to membrane stability during thermal stress [48,49], these associations are consistent with the possibility that microbial alterations and host metabolic adjustments represent coordinated responses to cold exposure. This pattern is also compatible with the concept of gut-liver interactions, in which intestinal microbial changes may be linked to host metabolic responses through metabolite-mediated communication [16,58].

However, these relationships should be interpreted cautiously. Pearson correlation analysis can identify co-variation, but it does not establish causality or directionality. Therefore, the present results do not demonstrate that gut microbial shifts directly drove hepatic metabolic changes. Rather, they indicate that intestinal microbial and hepatic metabolic changes were significantly associated under cold stress and may be connected within a gut–liver interaction framework. Because both the microbiota and metabolomic datasets were obtained from endpoint samples, future studies combining time-resolved sampling, targeted metabolite validation, and microbiota manipulation will be needed to clarify the mechanistic basis and causal direction of this relationship.

## 5. Conclusions

In summary, acute cold stress induced physiological and metabolic disturbances in *L. capito*, including hepatic oxidative imbalance, serum enzyme alterations, intestinal microbial dysbiosis, and changes in hepatic lipid- and energy-related metabolism. Correlation analysis further revealed significant associations between altered gut microbiota and hepatic metabolites, suggesting that intestinal microbial changes and hepatic metabolic responses may be linked under cold stress. However, these findings are association-based and do not establish causality. Overall, this study provides an integrated view of the biochemical, microbial, and metabolic responses of *L. capito* to acute low-temperature exposure.

## Figures and Tables

**Table 1:** Significantly altered metabolites in the liver of *L. capito* after cold stress.

Metabolites	Categories	FC	*p*-Value	VIP	Trend
PC(15:0/16:0)	Lipids	0.9584	0.0001779	1.6069	down
PC(15:0/18:1(11Z))	Lipids	0.9764	0.003734	1.0806	down
PC(16:0/16:0)	Lipids	0.9043	5.538 × 10^−5^	2.0949	down
LysoPC(20:2(11Z,14Z))	Lipids	1.0333	0.04619	1.1594	up
LysoPC(18:3(6Z,9Z,12Z))	Lipids	1.0319	0.0002827	1.3557	up
SM(d18:1/18:0)	Lipids	0.9748	0.03251	1.0506	down
Cytidine	Nucleic acids	0.8288	7.002 × 10^−7^	3.2465	down
Cytosine	Nucleic acids	0.9028	2.185 × 10^−9^	2.5357	down
Guanine	Nucleic acids	0.9796	0.00179	1.0656	down
ADP	Nucleic acids	1.0274	0.01904	1.0651	up
5′-CMP	Nucleic acids	1.0434	0.0001454	1.4709	up
Argininosuccinic acid	Peptides	1.0739	0.001305	1.8251	up
Cortisol	Steroids	1.0641	0.02897	1.2677	up
Ascorbic Acid	Vitamins and Cofactors	0.8989	0.003484	1.8044	down
D-Pantothenic acid	Vitamins and Cofactors	1.067	4.465 × 10^−5^	1.8811	up

VIP, variable importance in projection; FC, fold change calculated from the arithmetic mean values of each group. Metabolites with VIP > 1.0 and FDR-adjusted *p* < 0.05 were considered differential metabolites between the two groups. A total of 157 metabolites belonging to other classes are not shown.

## Data Availability

The original contributions presented in this study are included in the article. Further inquiries can be directed to the corresponding authors.
